# Safety of MRI in patients with retained cardiac leads

**DOI:** 10.1002/mrm.29116

**Published:** 2021-12-27

**Authors:** Bach T. Nguyen, Bhumi Bhusal, Amir Ali Rahsepar, Kate Fawcett, Stella Lin, Daniel S. Marks, Rod Passman, Donny Nieto, Richard Niemzcura, Laleh Golestanirad

**Affiliations:** ^1^ Department of Radiology, Feinberg School of Medicine Northwestern University Chicago Illinois USA; ^2^ Department of Electrophysiology, Feinberg School of Medicine Northwestern University Chicago Illinois USA; ^3^ Department of Biomedical Engineering, McCormick School of Engineering Northwestern University Evanston Illinois USA

**Keywords:** cardiovascular implantable electronic devices, finite element methods, fragmented retained leads, RF heating, safety, SAR

## Abstract

**Purpose:**

To evaluate the safety of MRI in patients with fragmented retained leads (FRLs) through numerical simulation and phantom experiments.

**Methods:**

Electromagnetic and thermal simulations were performed to determine the worst‐case RF heating of 10 patient‐derived FRL models during MRI at 1.5 T and 3 T and at imaging landmarks corresponding to head, chest, and abdomen. RF heating measurements were performed in phantoms implanted with reconstructed FRL models that produced highest heating in numerical simulations. The potential for unintended tissue stimulation was assessed through a conservative estimation of the electric field induced in the tissue due to gradient‐induced voltages developed along the length of FRLs.

**Results:**

In simulations under conservative approach, RF exposure at B_1_^+^ ≤ 2 µT generated cumulative equivalent minutes (CEM)_43_ < 40 at all imaging landmarks at both 1.5 T and 3 T, indicating no thermal damage for acquisition times (TAs) < 10 min. In experiments, the maximum temperature rise when FRLs were positioned at the location of maximum electric field exposure was measured to be 2.4°C at 3 T and 2.1°C at 1.5 T. Electric fields induced in the tissue due to gradient‐induced voltages remained below the threshold for cardiac tissue stimulation in all cases.

**Conclusions:**

Simulation and experimental results indicate that patients with FRLs can be scanned safely at both 1.5 T and 3 T with most clinical pulse sequences.

## 1 INTRODUCTION

With an aging population and the rising prevalence of cardiac disease, cardiovascular implantable electronic devices (CIEDs) are used more frequently. Today, there are more than 3 million Americans with CIEDs,^1^ and the number grows by 80 000 annually.^2^ It is estimated that 50%–75% of these patients may need MRI for cardiac or noncardiac indications,^3^ with many patients requiring repeated examination.^4^

Several studies have assessed the safety of MRI in patients with CIED leads connected to working devices. Some patients, however, require procedures that lead to “lead extraction”, leaving fractions of the device in situ, in which case the original safety studies of the intact device are not applicable. For example, patients undergoing heart transplant usually have a CIED in place, often with multiple leads in the vascular space.^5^ At the time of surgery, the physician attempts to remove the device by cutting the leads and extracting the pulse generator (leaving behind an exposed metal contact). Complete lead removal, however, is not always possible, especially when there is adhesion of leads to the vessel wall or significant calcification.^5^ This leaves a sizeable cohort of patients with fragmented retained leads (FRLs).^6^, ^7^ Magnetic resonance imaging is needed in most of these patients either for cardiac indications (e.g., rejection monitoring, perfusion assessment) or neurological and orthopedic exams. Today, however, there is no consensus on the safety of MRI in patients with retained cardiac leads. Although MR‐conditional CIEDs have been available since 2011, the conditional labeling of these devices applies only to the intact device and leads in their originally intended configuration. As such, studies assessing the MRI safety of pacemaker or implantable cardioverter defibrillator generators mostly exclude patients with FRLs.^8^, ^9^

Due to lack of guidelines, the decision on whether to perform MRI on a patient with a FRL falls upon the physician’s judgment, and is often based on his or her assessment of risk‐to‐benefit ratio. The goal of this work is to provide a comprehensive assessment of MRI hazards in patients with FRLs, to provide actionable information to clinicians encountering such cases. We expanded our previous work, which assessed specific absorption rate (SAR) amplification around patient‐derived FRL models in a single homogenous body model^10^ by performing a thorough search for the worst‐case heating scenario in body models with 30 unique combination of permittivity and conductivity covering a range of low to high values reported for biological tissues. Additionally, we included new FRL models with folded trajectories and double leads in close proximity, as these topologies are shown to alter RF heating.^11^ To provide action points for clinicians, a conservative estimation of CEM_43_ for different exposure times and B_1_^+^ values was provided along with thresholds of tissue thermal damage. We also performed experiments measuring RF heating around fragments of commercial CIED leads implanted in gel phantoms to gain additional confidence as to typical level of RF heating around FRLs in vitro. Finally, we assessed other sources of MRI hazard including the potential for unintended tissue stimulation.

## 2 METHODS

### 2.1 Sources of MRI hazard in patients with FRLs

The technical specification ISO‐TS 10974 describes known sources of potential hazardous interaction between MRI fields and an active implantable medical device. These include risk of gradient‐induced and RF‐induced device heating, potential harm due to gradient‐induced vibration, gradient‐induced extrinsic potentials, and gradient‐induced device malfunction, as well as risks associated with B_0_‐inserted force and torque.^12^

Gradient‐induced device heating is the result of time‐varying imaging gradient dB/dt, inducing eddy currents on active implantable medical device conductive surfaces such as enclosures, battery components, and circuit traces. For active implantable medical devices with extended leads that do not contain larger conductive surfaces, there is no known mechanism for MRI‐induced eddy current heating to occur in the lead (ISO‐TS 10974:9). Therefore, this is not a source of concern in patients with FRLs.

Gradient‐induced eddy currents also generate a time‐varying magnetic moment that interacts with the static magnetic field (B_0_), causing vibration of conductive surfaces (and subsequently the device), which could lead to device malfunction (ISO‐TS 10974:10). For the same reasons explained previously, this is not a source of concern in patients with FRLs. Finally, the risks associated with lead traction and dislodgment due to B_0_‐inserted force and torque are negligible, as CIED leads are composed of nonmagnetic material. Radiofrequency‐induced heating and gradient‐induced extrinsic voltages are therefore the only potential sources of MRI hazard in patients with FRLs.

### 2.2 Radiofrequency heating

Radiofrequency heating is due to the antenna effect, in which the electric field of MRI transmit coil couples with conductive leads and amplifies the SAR of RF energy in the tissue, usually occurring around the tip of an elongated implant.^10^, ^13^, ^14^, ^15^, ^16^, ^17^ Temperature rises up to Δ*T* = 30°C have been reported at tips of abandoned cardiac leads in phantom experiments at 1.5 T.^18^, ^19^

Magnetic resonance imaging RF heating of an elongated lead is known to be a resonance phenomenon, depending on lead’s length and the distribution of MRI electric field’s phase along lead’s trajectory.^13^, ^20^, ^21^ We created clinically relevant FRL models based on medical images of 10 representative patients, including trajectories with folds or multiple leads in close proximity, as they may alter RF heating.^19^, ^22^, ^23^, ^24^

Another important factor influencing RF heating is the electrical property of the medium surrounding the lead.^25^, ^26^, ^27^, ^28^ To determine the worst‐case scenario, we performed numerical simulations with each FRL model registered to homogenous body models with 30 unique combinations of permittivity and conductivity covering a range of low to high values reported for biological tissues (σ∈[0.1, 1]*S*/*m*, ε*_r_* ∈[40, 80]). The rationale for using a homogenous body model with varying electrical properties was based on a recent study that showed such a model can predict the worst‐case SAR generated in a heterogeneous body mode at a reduced computational cost.^29^ Finally, the position of the lead within the MRI RF coil affects its RF heating.^30^, ^31^ Thus, RF heating was assessed for each FRL+ body model positioned inside the MRI body coil at landmarks corresponding to head, chest, and abdomen imaging.

Temperature rise in the tissue was conservatively estimated by solving the simplified Penne’s bio‐heat equation, excluding cooling effects of the perfusion:

$$c\rho \frac{\partial T}{\partial t}‐\nabla k\nabla T=\rho \left(\text{SAR}\right)$$

where *T* is the temperature; *ρ* is the density (1000 kg/m^3^); *c* is the specific heat capacity of the tissue (4150 J kg^−1^℃^−1^); and *k* is the isotropic thermal conductivity (0.42 W/m^−1^℃^−1^).^32^ For each FRL model, thermal simulations were performed in the human body model that generated the highest local SAR at the corresponding imaging landmark (abdomen, chest, and head), with the input power of coils adjusted to produce B_1_^+^ in a range of 1 to 5 µT. The selected B_1_^+^ values cover the whole range of routine clinical protocols including high‐SAR sequences.

To provide actionable information, we calculated the worst‐case cumulative equivalent minutes at 43°C (CEM_43_) for a range of exposure times (1–10 min) and B_1_^+^ values (1–5 µT) at 1.5 T and 3 T. CEM_43_ is currently the accepted metric for thermal dose assessment that correlates well with thermal damage in variety of tissues.^33^, ^34^ The calculation of CEM_43_ requires knowledge of thermal history, as:

$${\text{CEM}}_{43}=\Delta t\times R^{\left(43‐T\right)}$$

where Δ*t* indicates integration over the length of exposure; *T* is the average temperature during the exposure time; and *R* is a constant equal to 0.25 for *T* < 43°C and 0.5 for *T* > 43°C.^33^ To be conservative, CEM_43_ was calculated using temperature rise Δ*T* from the FRL model that generated the maximum heating. Finally, we performed experiments with fragments of a commercial cardiac lead implanted in a gel phantom during RF exposure at 1.5 T and 3 T to gain additional confidence as to level of RF heating observed in vitro.

### 2.3 Gradient‐induced voltage

Extrinsic electric field potentials are gradient‐induced voltages that develop between spatially separated electrodes within a single lead or between electrodes of a multilead system (ISO‐TS 10974:13). If the FRL is in contact with the excitable tissue, these voltages could potentially cause unintended stimulation. We assessed the possibility of unintended tissue stimulation due to gradient‐induced extrinsic potentials along FRLs based on a conservative estimation of electric potentials developed along the length of FRL models as described in ISO‐TS 10974:13. These voltage values were then used to calculate a conservative estimation of electric field E induced in the tissue developed between the two ends of each FRL, and the potential for unintended tissue stimulation was assessed in the context of cardiac stimulation thresholds.^35^, ^36^

### 2.4 Patient‐derived FRL models

Chest CT and X‐ray images of 100 patients with a history of implanted cardiac devices who had been admitted to Northwestern Memorial Hospital between 2006 and 2018 were obtained through a search of the Northwestern Universities’ Enterprise Data Warehouse. Images were inspected by a radiologist (A.R.) for the presence of FRLs. From patients identified with FRL, 10 patients who had both chest X‐ray and CT images that clearly delineated FRL trajectory and topology were included in the study. Patient characteristics are given in Table 1. Use of imaging data for the purpose of simulation and modeling was approved by Northwestern Memorial Hospital’s ethics review boards.

**TABLE 1:** Characteristics of patients with cardiovascular implantable electronic device fragmented retain leads

Patient ID#	Age	Sex	FRL location	Device type and manufacturer	Lead model(s)	Date of implantation (month/year)	Date of extraction (month/year)	Apparent/True length of FRL (cm)
FRL1	24	M	Left subclavian vein	Medtronic dual‐chamber ICD	Capsurefix Novus 5076–RA	9/2007	1/2013	13.4 & 10.5
			SVC		Sprint Fidelis 6931–RV			67.9 &10.5
FRL2	56	M	SVC/RA junction	Medtronic biventricular ICD	Capsurefix Novus 5076–RA	5/2002	04/2007	14.2
			Left subclavian vein		Sprint Quattro 6944–RV 4193 Attain OTW–LV	9/2005–LV		90.4
FRL3	52	F	SVC	Boston Scientific dual‐chamber ICD	1688 TC–RA Reliance G 0185–RV	1/2007	08/2011	8.9 & 4.4
			Left subclavian vein	Abbott St Jude RA lead				13.3 & 6.5
FRL4	64	F	SVC/RA junction	Medtronic biventricular ICD	Capsurefix 5076–RA	4/2007	7/2008	18.0
					Attain OTW–LV			41.2
					Sprint Fidelis–RV			
FRL5	66	F	SVC/RA	Medtronic single‐chamber ICD	Sprint Quattro 6947‐65–RV	4/2012	9/2012	17.2
			Left subclavian vein					107.3
FRL6	57	M	SVC	Medtronic biventricular ICD	Capsurefix Novus 5076–RA	4/2003	12/2009	15.5
			Left subclavian vein		Sprint Quattro 6947–RV	9/2008–LV		54.3
					Attain Starfix 4195–LV			
FRL7	50	M	SVC/RA junction	Medtronic biventricular ICD	Capsurefix Novus 5076–RA	1/2006	6/29/2015	2.0
					Attain OTW 4194–LV	3/2011–LV		26.4
					Sprint Quattro 6947–RV	11/2011–RV		
FRL8	57	M	SVC/RA	Medtronic biventricular ICD	5071–LV	8/2012	05/2013	4.5
					Capsurefix Novus 5076–RA			46.9
					6947–RV			
FRL9	64	M	SVC	Boston Scientific dual‐chamber ICD	Boston Scientific 4052–RA	6/2003	11/2005	12.7 & 12.7
			Left subclavian vein		Guidant Endotak Reliance 0185–RV			21.8 & 18.1
FRL10	29	M	SVC	Medtronic biventricular ICD	Capsurefix Novus 5076–RA	2/2005	08/2015	8.5
			Left subclavian vein		Sprint Fidelis 6949–RV	10/2009–LV		96.0
					Attain Ability 4196–LV			

Abbreviations: ICD, implantable cardioverter defibrillator; LV, left ventricle; RA, right atrium; RV, right ventricle; SVC, super vena cava.

**FIGURE 1:**
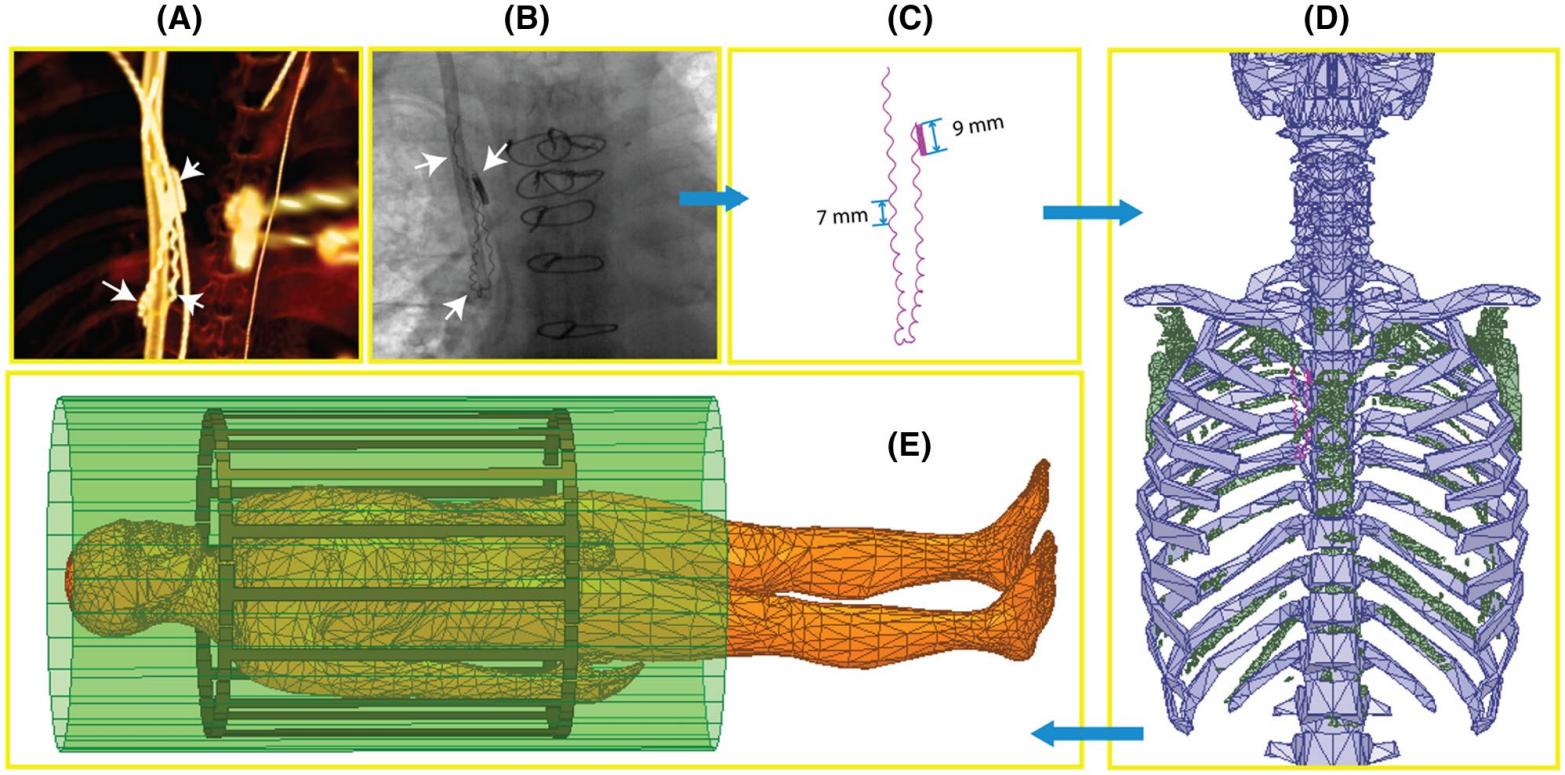
Steps of image segmentation and fragmented retained lead (FRL) model construction. (A–C) Computed tomography images were used to extract the 3D trajectory of FRL, whereas X‐ray images were used to reconstruct the FRL’s structure (e.g., number and pitch of loops). White arrows show the FRL on each image. (D) A triangulated surface of patient’s ribcage (green) was created and aligned with the ANSYS multicompartment body model (blue) to position the patient‐derived FRL model inside the ANSYS human body model. (E) Body model positioned inside the MRI coil

Lead trajectories were semi‐automatically segmented from CT images using Amira (Amira 5.3.3; FEI, Valley City, ND) by applying a thresholding algorithm based on an intensity histogram analysis, which extracted a preliminary mask of the hyperdense lead from the CT image (Figure 1A). Lead centerlines were manually extracted and exported to a CAD tool (Rhino 3D; Robert McNeal and Associates, Seattle, WA), where 3D models of FRLs were constructed around them. The CIED leads usually include individual coiled wires extending from the proximal to the distal end of the lead. The topology of these coiled wires changes during the extraction process as the physician applies force to extract the lead due to surrounding adhesions. We used X‐ray images to reconstruct the details of the FRL structure, including the number of microloops and the variation in their pitch (Figure 1B,C). To correctly position the FRLs in the human body model, we created a triangulated surface of each patient’s ribcage from CT images, which was then used to align and coregister the FRL of that patient to the ANSYS multi‐compartment human body model^37^ (Figure 1D). Once the FRL was in its correct position, we merged different body tissues to create a homogenous body model that was then assigned to a range of different electrical conductivity (σ = 0.1, 0.2, 0.4, 0.6, 0.8, and 1 *S/m*) and permittivity (ε*_r_* = 40, 50, 60, 70, and 80) to cover all relevant biological values.^38^ This is reasonable, as a recent study has shown a homogeneous body model with conductivities varied in a range of low to high values (0.01 *S/m →* 1 *S/m*) can predict the full range of SAR variations predicted by a heterogeneous body model.^29^

### 2.5 Magnetic resonance imaging coils

Numerical models of two high‐pass birdcage body coils (620 mm length, 607 mm diameter) were implemented in ANSYS Electronic Desktop and tuned to their respective Larmor frequencies: 64 MHz (1.5 T) and 127 MHz (3 T). Each RF coil consisted of 16 rungs connected at each end to two end rings and shielded by an open cylinder (1220 mm length, 660 mm diameter). The coils and shields were made of copper, and their dimensions were chosen based on a typical clinical body coil. A quadrature excitation was implemented by feeding the coils at two ports on the bottom end ring that were 90° apart in position and phase. Coils were tuned by lumped capacitors distributed at the end‐ring gaps (86.5 pF for 64 MHz and 17 pF for 127 MHz) and matched to 50 Ω using a single capacitor at each port (100 pF at 64 MHz and 45.6 pF at 127 MHz) in series with an ideal voltage source with a 50‐Ω internal resistance.

### 2.6 Specific absorption rate and thermal simulations

**FIGURE 2:**
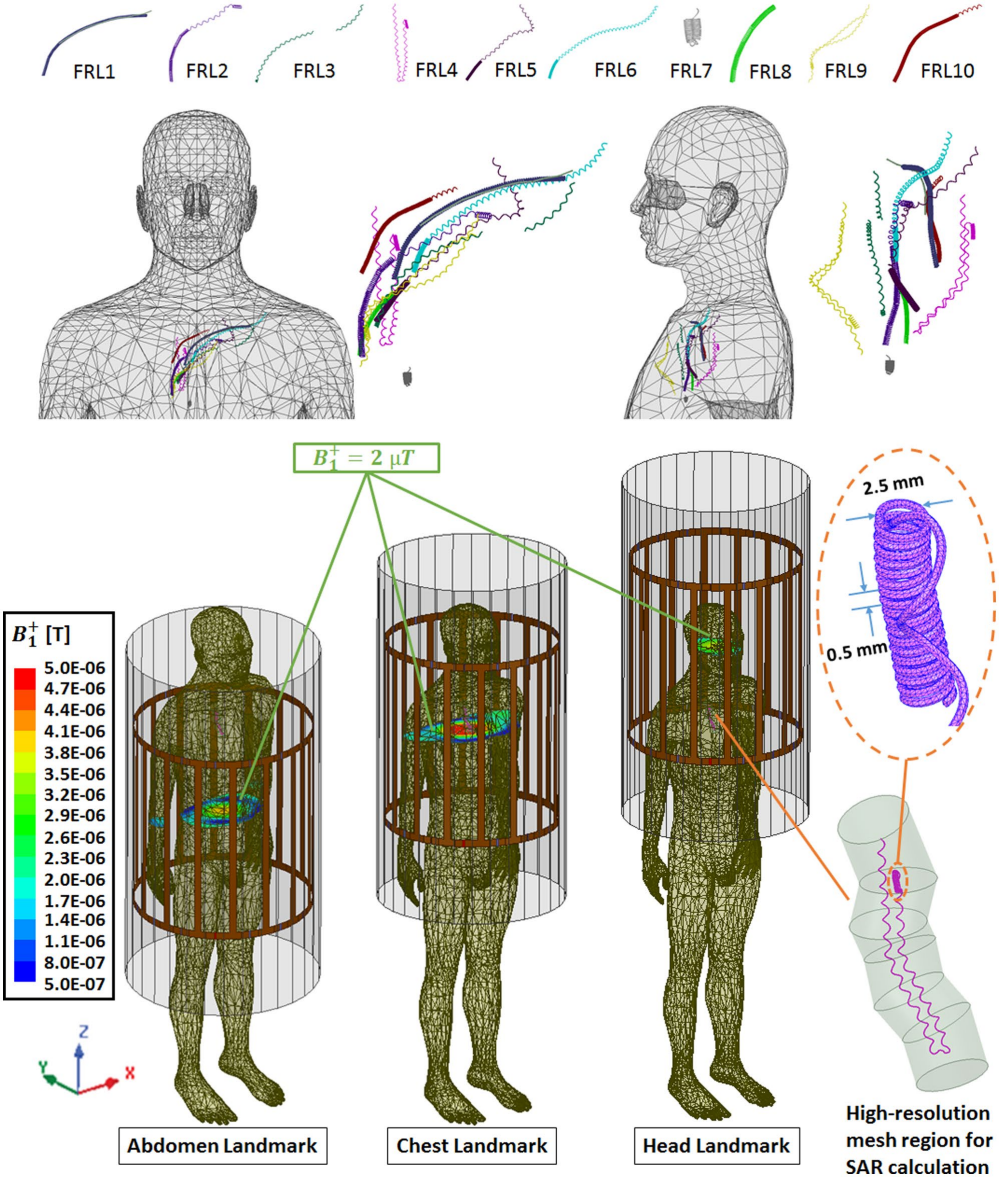
Top row: Trajectories and detailed structures of FRL models extracted from 10 patients (namely FRL1‐FRL10). FRL 4 and 7 represent folded trajectories. FRL 9 was a case of a patient with two closely situated retained leads. Middle row: Front and side views of reconstructed FRLs and their relative locations in the human body model. Bottom row: Position of the body model inside the MRI coil at different imaging landmarks (i.e., abdomen, chest, and head). The high‐resolution mesh area around the lead in which 1*g* SAR was calculated is also shown

A total of 1800 numerical simulations were performed, with 10 realistic FRL models incorporated in body models with 30 unique combinations of electrical conductivity and permittivity, positioned in two MRI body coils (1.5 T and 3 T) at three different imaging landmarks (abdomen, chest, and head). The FRL wire diameters ranged from 0.35 to 0.6 mm and were assigned as titanium for the simulation. The maximum value of 1*g*‐averaged SAR (referred to as MaxSAR1g) was calculated inside a conformal cylindrical volume (diameter = 2 or 3 cm depending on the shape of the FRL) that surrounded the FRL as illustrated in Figure 2.

To ensure good numerical convergence, the initial mesh was set such that the maximum element size was <2 mm on the FRL, <2 mm in the surrounding conformal region in which MaxSAR1g was calculated, and <50 mm in the body. The ANSYS HFSS was set to follow an adaptive mesh scheme with successive refinement of the initial mesh between iterative passes until the difference in magnitude of the S‐parameters fell below a set threshold of 0.02. Total time taken for each simulation was from 30 min to two and half hours (depending on the length and the shape of FRL) on a Dell server with 1.5 TB memory and 2_Xenon Gold 6140 CPUs each having 18 processing cores. Table 2 gives mesh statistics for a typical simulation.

**TABLE 2:** Details of mesh statistics for the simulation of FRL4 at 3 T for the abdomen imaging landmark

Parts	Num tets	Min edge length (mm)	Max edge length (mm)	RMS edge length (mm)	Min tet. vol. (mm^3^)	Max tet. vol. (mm^3^)	Mean tet. vol. (mm^3^)	SD vol. (mm^3^)
Lead	22 597	0.0538	0.7218	0.4354	7.9218^‐08^	9.5171^‐03^	1.8853^‐03^	8.6679^‐04^
SAR region	50 6346	0.0566	2.3516	1.5475	7.7631^‐08^	7.6573^‐01^	1.7217^‐01^	1.0858^‐01^
Body	89 169	1.0457	56.6824	24.927	8.3179^‐03^	9.7123^03^	8.7479^02^	1.2956^03^

Thermal simulations were performed in the human body model that produced the highest local SAR at each imaging landmark (abdomen, chest, and head), with input power of coils adjusted to produce a spatial mean of B_1_^+^ amplitude (i.e., complex magnitude of B_1_^+^ averaged over an axial plane passing through the isocenter of the coil; Figure 2) in a range of 1 to 5 µT.

### 2.7 Radiofrequency heating measurements

**FIGURE 3:**
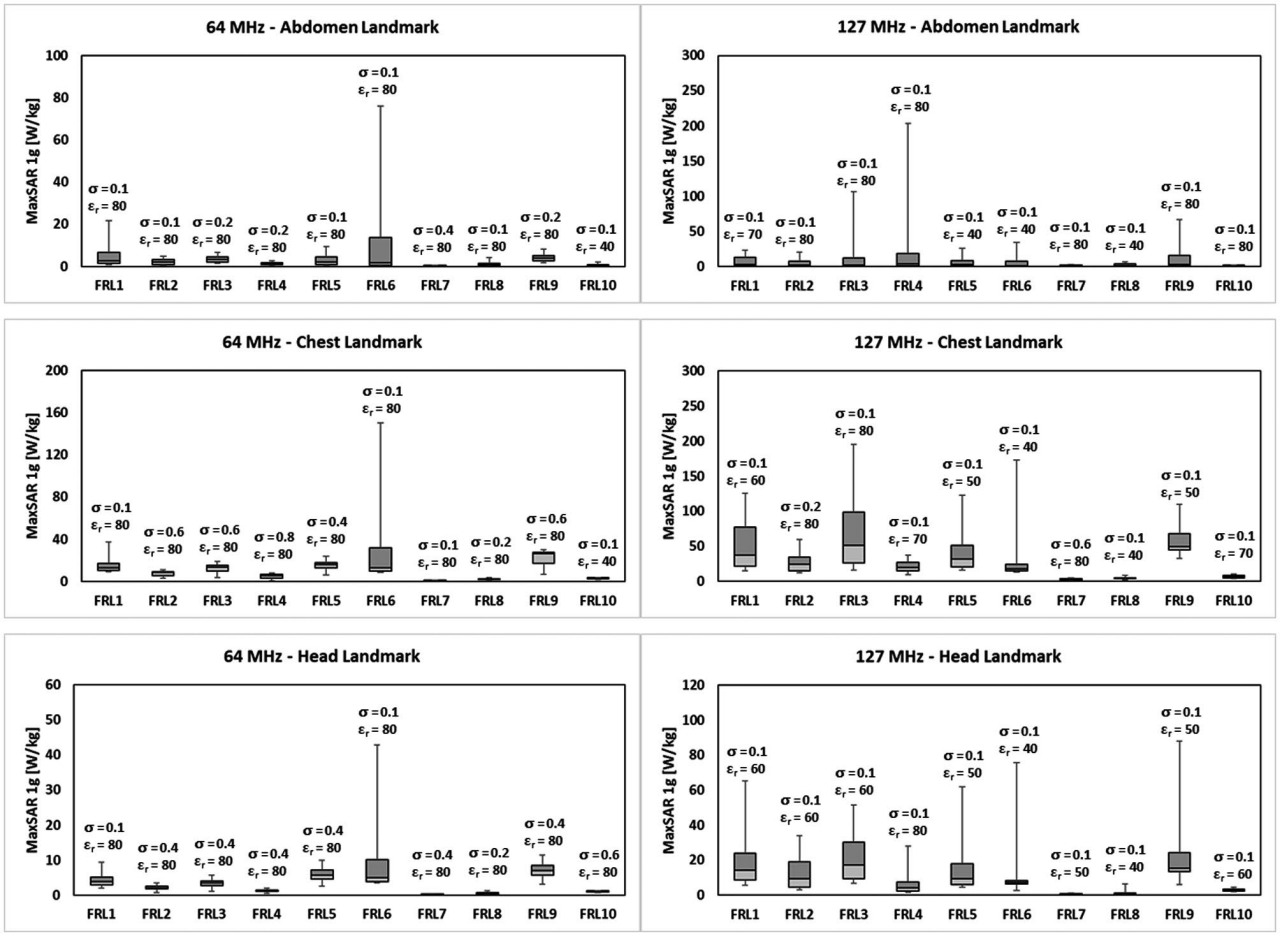
Comparison of maximum value of 1*g*‐averaged specific absorption rate (MaxSAR1g) of all leads for each combination of landmark position and frequency. For each simulation, the input power of the coil was adjusted such that the spatial mean of the complex magnitude of B_1_^+^ (i.e., 1/2 ‖B_1x_ + jB_1y_‖) was 2 μT on an axial plane passing through the iso‐center of the coil

To gain additional confidence as to typical values of RF heating occurring in the tissue surrounding FRLs, we performed phantom experiments with four FRL models reconstructed from commercially available pacemaker leads (Medtronic 5076; diameter of internal wires 0.1 mm), with trajectories mimicking those that generated highest temperature rise in simulations. Specifically, we reconstructed FRL #6, which generated highest temperature rise at 1.5 T for all three landmarks, and FRL models #4, #3 and #9, which generated highest temperature rise at 3 T at abdomen, chest and head landmarks, respectively (see Figure 3).

Experiments were performed in a Siemens Aera 1.5T scanner and a Siemens Prisma 3T scanner (Siemens Healthineers, Erlangen, Germany). The FRL models were positioned inside a human‐shaped container filled with a gel (12‐cm thick) prepared by mixing 10 g/L of Polyacrylic Acid (Sigma‐Aldrich, St. Louis, MO) and 1.32 g/L sodium chloride with distilled water. The anthropomorphic phantom was designed using segmentation of patient images as described in our previous work.^27^ Bulk conductivity and relative permittivity of the gel was measured to be σ = 0.46 *S*/*m* and ε*_r_* = 87 using a dielectric probe kit (85070E; Agilent Technologies, Santa Clara, CA) and a network analyzer.

RF heating was measured for two scenarios. First, FRL models were positioned in a location analogous to the middle of the chest, similar to what we observed in patient images, namely position P1 as illustrated in Figure 6A. Second, and to be more conservative, we also measured temperature rise for FRL models in a high field exposure location, namely position P2 in Figure 6A, which was near the phantom wall. To find the location of maximum field exposure, we performed experiments with a simple insulated copper wire (Figure S1), which was orientated parallel to the phantom’s long walls at three different depths from the gel’s surface. The temperature profile showed a right–left asymmetry in local E‐field distribution, which was in agreement with the earlier findings.^39^ The highest temperature rise occurred when the wire was located along the phantom’s left wall and 2 cm from the gel’s surface. This location was chosen as P2 to position the FRL at the maximum E‐field exposure. Two fluoroptic temperature probes (OSENSA, Burnaby, BC, Canada) were secured at both tips for all four FRL models. Temperature rise ∆*T* in the gel was recorded during 10 minutes of RF exposure using a high‐SAR T_1_ turbo spin‐echo sequence (TE = 7.3 ms, TR = 814 ms, flip angle = 150° for 1.5T scans and TE = 7.5 ms, TR = 1450 ms, flip angle = 150° for 3T scans). An additional temperature probe was also positioned inside the phantom far from the implant position, to observe the background heating (Figure 6A). The sequence parameters were adjusted to generate the RMS B_1_^+^ of 2 µT in the phantom. Imaging was performed using the scanner’s built‐in body coil. For each FRL model, the phantom was positioned alternately such that the abdomen/chest/head of the phantom was at the coil’s iso‐center. For all experiments, precise positioning of the FRLs inside the gel was ensured by using the support pillars with adjustable height, which were positioned on a gird fitted to the bottom surface of the anthropomorphic phantom. Figure 6A shows the details of the experimental setup.

### 2.8 Estimation of gradient‐induced extrinsic voltages

To obtain a conservative estimation of electric field induced in the tissue, we first calculated the injected *V*_emf_ along each FRL using the Tier 1 approach described in ISO‐TS10974:13.^12^ This approach is based on simulated values of electric fields induced on the surface of a cylinder of 20‐cm radius by a dB/dt = 100 T/s, and gives the most conservative estimation of extrinsic voltages for leads with length <63 cm. We then estimated the electric‐field E induced in the tissue between the two ends of each FRL by dividing the *V*_emf_ by the distance between the end tips (Figure S2). These values were compared against cardiac‐stimulation thresholds provided in the literature.^40^, ^41^, ^42^

## 3 RESULTS

### 3.1 Specific absorption rate distribution and maximum temperature rise in the tissue

Figure 3 gives MaxSAR1g values for each FRL model during RF exposure at 64 MHz (1.5 T) and 127 MH (3 T) and for the body model positioned at head, chest, and abdomen landmarks. At each imaging landmark, the input power of the coil was adjusted such that the spatial mean of B_1_^+^ amplitude was 2 µT at the coil’s iso‐center. Boxcars represent the variation of MaxSAR1g as a function of electrical properties (σ and ε*_r_*) of the body model. As it can be observed, the specific combination of tissue permittivity and conductivity that produced maximum SAR was different for each FRL model, emphasizing the interdependency of different factors affecting RF heating.^24^ Plots of variation of MaxSAR1g as a function of conductivity and permittivity are found in Supporting Information Figures S3–S7. In general, lower values of conductivity generated higher SAR in most models, with the trend being substantially more pronounced at 3 T.

MaxSAR1g (mean ± SD) was 3.50 ± 4.23 W/kg for the head imaging landmark, 11.34 ± 14.20 W/kg for the chest imaging landmark, and 3.20 ± 6.56 W/kg for the abdomen imaging landmark for RF exposure at 1.5 T (averaged over all body compositions and all lead models; *N* = 300). MaxSAR1g was significantly higher at chest imaging landmark compared with both head landmark and abdomen landmark (one‐tail *t*‐test; *p*‐value < 1^−04^). There was no significant difference between MaxSAR1g at head and abdomen landmarks (two‐tail *t*‐test; *p*‐value = .5).

At 3 T, MaxSAR1g was 11.79 ± 14.52 W/kg for the head imaging landmark, 32.09 ± 34.65 W/kg for the chest imaging landmark, and 6.83 ± 17.92 W/kg for the abdomen imaging landmark (averaged over all body compositions and all lead models; *N* = 300). MaxSAR1g was significantly higher at chest imaging landmark compared with both head landmark and abdomen landmark (one‐tail *t*‐test; *p*‐value < 1^−04^). MaxSAR1g at head landmark was slightly higher MaxSAR1g at abdomen landmark (one‐tail *t*‐test; *p*‐value = 1^−04^). At all imaging landmarks, MaxSAR1g was significantly higher at 3 T compared with 1.5 T (one‐tail paired *t*‐test; *p*‐values < 1^−04^).

**FIGURE 4:**
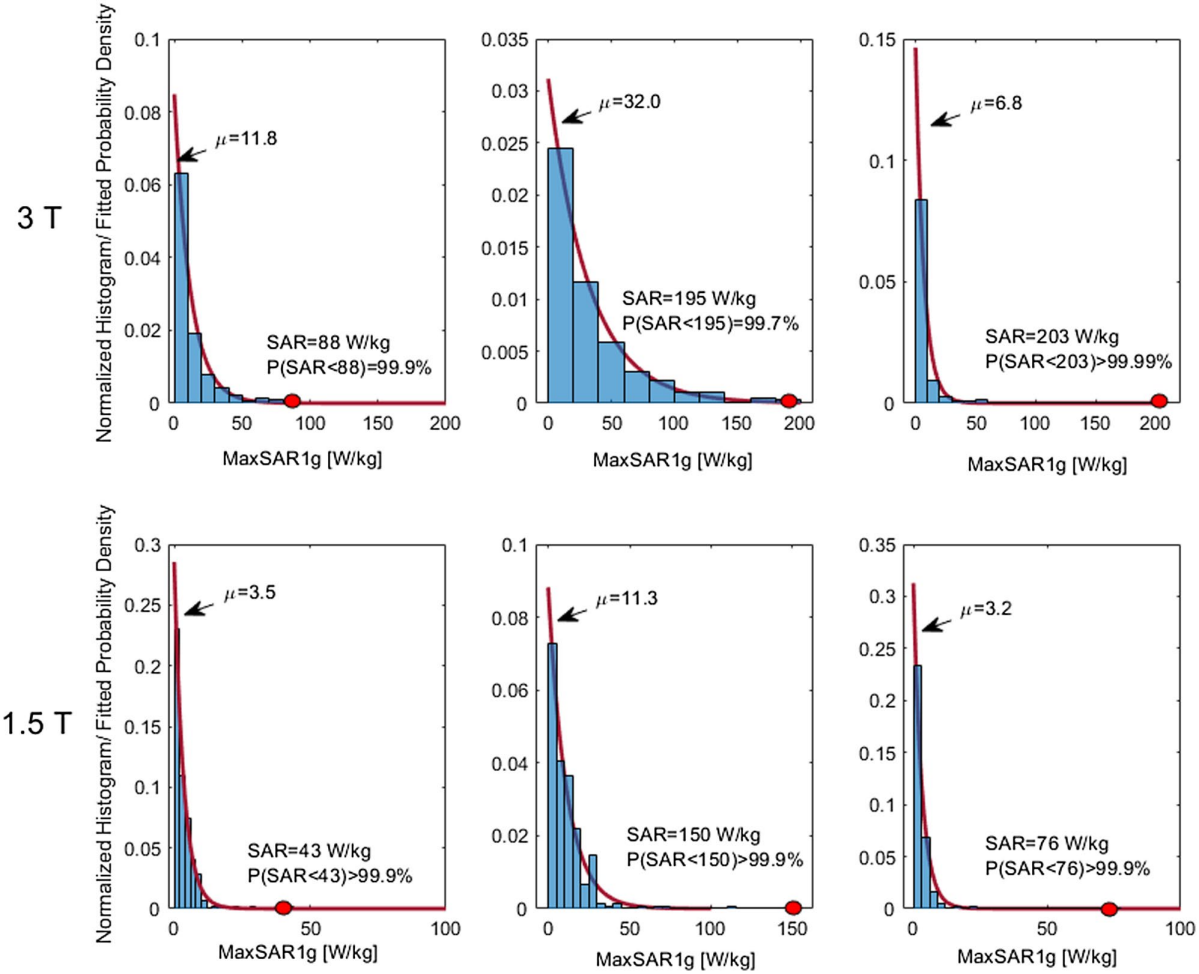
Normalized specific absorption rate (SAR) histograms pooling *N* = 300 body models and FRL leads for each field strength and at each imaging landmark. An exponential probability density function (mean = μ) was fitted to the SAR distribution for each case. Red markers show the location of extreme cases associated with the body + FRL model that created the maximum SAR. The value of the maximum SAR and the probability of an individual SAR being less than this value are also given

Figure 4 shows histograms of MaxSAR1g distribution at each field strength and for each imaging landmark. An exponential probability density function (mean of µ) was fitted to each distribution using the *MATLAB* and Statistics Toolbox Release 2019a (The MathWorks, Natick, MA). For each case, the model that created the most extreme data point on the SAR distribution was used for subsequent thermal analyses. Figure 4 shows that the approach was conservative, as >99% of cases generated SAR values below this limit.

### 3.2 Thermal dose

The temperature rise in the tissue after 10 min of continuous RF exposure at 1.5 T and 3 T is given in Tables S1 and S2, respectively. To be conservative, thermal simulations were performed using the body model with electrical properties that generated the highest MaxSAR1g.

**FIGURE 5:**
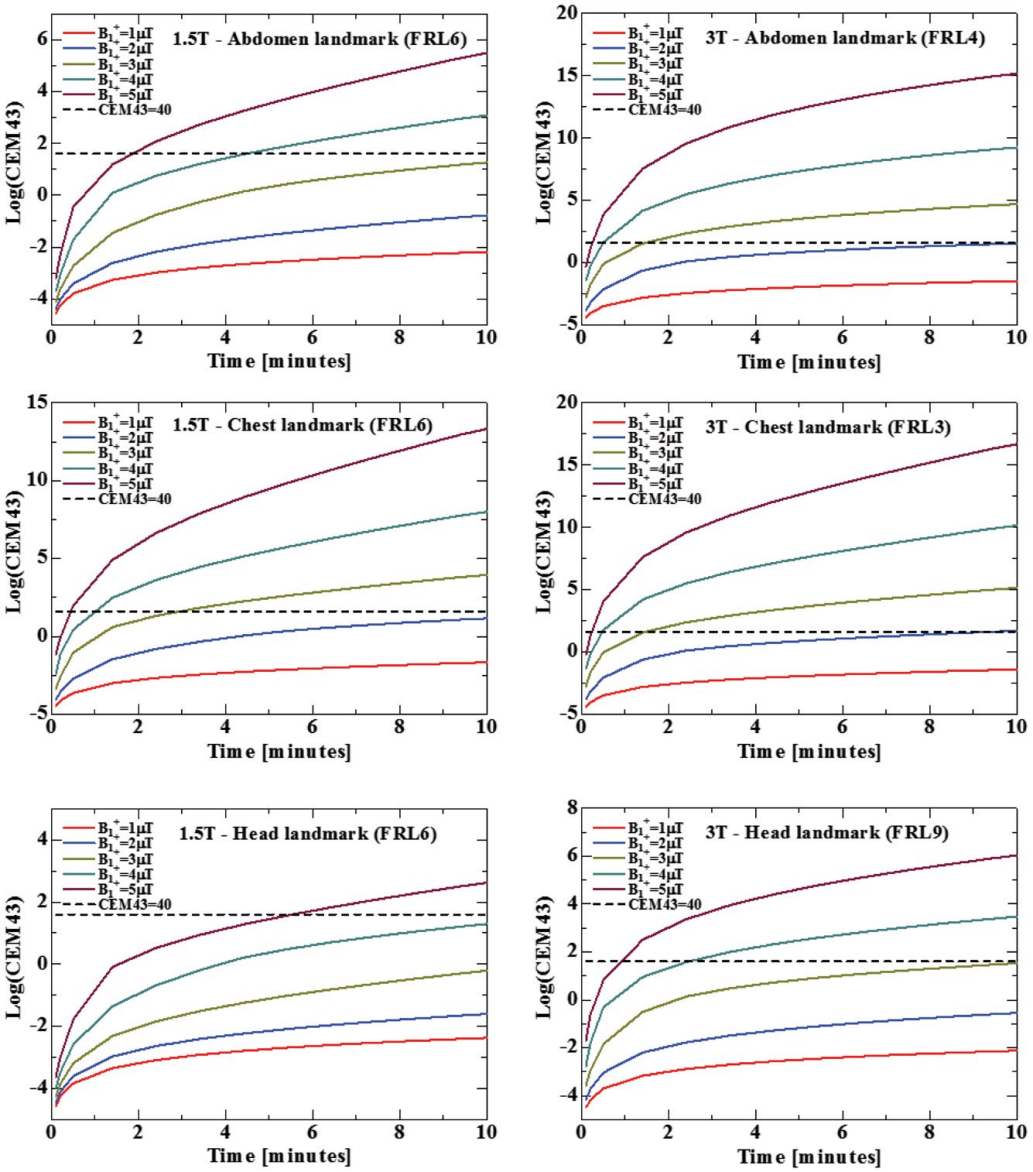
Cumulative equivalent minute (CEM_43_) values calculated at different B_1_^+^ levels as a function of acquisition time. The CEM_43_ values are calculated for the FRL model that generated the maximum temperature rise at each imaging landmark and RF frequency. Reported B_1_^+^ values are spatial means of the complex magnitude of the B_1_^+^ calculated on an axial plane passing through the coil’s iso‐center

At each imaging landmark and for each B_1_^+^ value, we calculated CEM_43_ corresponding to the FRL model that generated the highest RF heating (Figure 5). A review of published data on tissue damage due to thermal exposure can be found in Yarmolenko et al.^34^ For muscle tissue, CEM_43_ > 80 min has been reported to cause chronic damage, whereas 41 < CEM_43_ < 80 min was associated with acute but minor damages. From Figure 5 it can be observed that for B_1_^+^ ≤ 2 µT, RF exposure at both 1.5 T and 3 T generated CEM_43_ < 40 at all imaging landmarks, indicating no thermal damage for acquisition times < 10 minutes.

It should be noted, however, that calculated CEM_43_ values for continuous RF exposure are highly conservative, as MRI sequences typically have duty cycles much below 100%. Table 3 gives examples of typical clinical sequences for neuroimaging, cardiac imaging, and body imaging with their corresponding B_1_^+^ and acquisition times. The sequence‐specific CEM_43_ calculated for the FRL model that generated the maximum SAR is also given. As it can be observed that CEM_43_ remained well below 40 for all sequences, indicating no thermal damage.

**TABLE 3:** Sequence parameters and scanner‐reported RMS B_1_^+^ values for different imaging protocols on 1.5 T and 3 T scanners

1.5 T imaging						
Protocol	TE (ms)	TR (ms)	TA (min)	FA (°)	B_1_^+^ (µT)	CEM_43_
*Head landmark*						
SE T1 SAG	10	450	2:09	90	3.54	3.39^‐02^
AX DIFFUSION	89	7900	2:16	–	1.3	9.37^‐04^
AX FLAIR	86	9000	3:02	150	2	3.09^‐03^
AX GRE T2	25	830	1:58	20	0.3	4.93^‐04^
AX SWI	40	49	3:38	15	0.3	8.32^‐04^
AX T1 SE	12	450	2:26	150	3.04	1.40^‐02^
AX T2 TSE	94	3800	2:22	150	3.71	7.20^‐02^

*Chest landmark*						
COR TRUFI	1.21	491.33	0:20	64	4.01	3.92^‐01^
AX TRUFI	1.18	328.38	0:13	64	3.99	7.19^‐02^
COR VIBE	2.39, 4.77	6.63	0:22	10	1.28	2.08^‐04^
AX VIBE	2.39, 4.77	6.9	0:20	10	1.49	2.44^‐04^
TRUFI CINE 2C	1.16	38.22	0:06	60	3.98	2.20^‐03^
PC 3C IP AORTA	2.47	37.12	0:10	20	0.97	5.27^‐05^
PC RVOT IP	2.47	37.12	0:10	20	0.97	5.27^‐05^
PC TP AV	2.47	37.12	0:10	20	0.97	5.27^‐05^
TRUFI CINE SA	1.16	40.96	1:00	60	3.99	3.71^+01^
TFL PSIR	3.22	700	0:14	25	0.63	6.65^‐05^
*Abdomen landmark*						
T2 HASTE COR MBH	91	1400	0:48	180	4.2	1.73^‐01^
T2 HASTE FS TRA MBH	94	1400	0:05	160	3.1	4.70^‐05^
T2 BLADE FS TRA	91	2200	2:35	160	3.35	8.14^‐01^
T2 TSE FS TRA MBH	86	4780	0:54	160	3.77	6.57^‐02^
EP2D DIFF	54	6200	3:18	–	1.3	2.33^‐03^

3 T imaging						
Protocol	TE (ms)	TR (ms)	TA (min)	FA (°)	B_1_^+^ (µT)	CEM_43_
*Head landmark*						
T1‐TSE DARK‐FLUID	8.5	2000	4:38	150	1.34	6.21^‐03^
T1‐SPACE	9	700	6:41	–	1.81	5.32^‐02^
T1‐FL2D‐TRA	2.49	250	1:19	70	1.97	4.47^‐03^
T1‐FL2D_COR	2.49	250	1:19	70	2.25	1.04^‐02^
T2‐TSE‐TRA	100	6000	2:44	150	1.99	1.90^‐02^
T2‐TSE‐DARK‐FLUID	83	9000	4:14	150	1.46	7.52^‐03^
EP2D‐DIFF	81	3700	0:57	–	1.51	8.98^‐04^
TOF‐FL3D	3.42	21	5:33	18	2.02	8.81^‐02^
T1‐SPACE‐FS	10	750	7:00	–	2.15	2.65^‐01^
*Chest landmark*						
T1‐TSE‐DB	27	700	0:09	180	1.55	1.17^‐04^
T2‐TSE‐DB	71	800	0:05	180	1.85	6.42^‐05^
T2‐TSE‐DB‐FAST‐HR	70	600	0:04	180	2.2	6.78^‐05^
T2‐TRIM‐DB‐SAX	44	800	0:06	180	1.98	1.10^‐04^
T2‐TSE‐BLADE‐DB	76	750	2:05	180	1.19	5.54^‐03^
HASTE‐16‐SL‐DB‐PACE	49	750	1:20	160	1.39	5.46^‐03^
TRUFI‐SINGLESHOT	1.23	224.2	0:12	60	1.95	4.73^‐04^
DE‐OVERVIEW‐TFI‐PSIR	1.09	700	0:18	55	1.6	4.78^‐04^
*Abdomen landmark*						
T2‐HASTE‐COR‐MBH	87	1400	0:48	160	2.27	3.91^‐01^
T2‐TSE‐FS‐TRA	100	2200	3:25	160	2.04	3.30^+00^
T2‐BLADE‐FS‐TRA	89	2500	3:30	135	2.48	1.10^+01^
EP2D‐DIFF‐TRA	39	4600	3:28	–	1.89	1.47^+00^
T1‐VIBE‐DIXON‐TRA	1.29, 2.52	3.97	0:15	9	1.52	2.64^‐04^
T2‐SPACE‐COR	701	2400	4:46	120	2.1	1.01^+01^

Abbreviations: FA, flip angle; GRE, gradient echo; IP, immunoprecipitation; SE, spin echo; TA, acquisition time; TSE, turbo spin echo.

### 3.3 Experimental measurements

**FIGURE 6:**
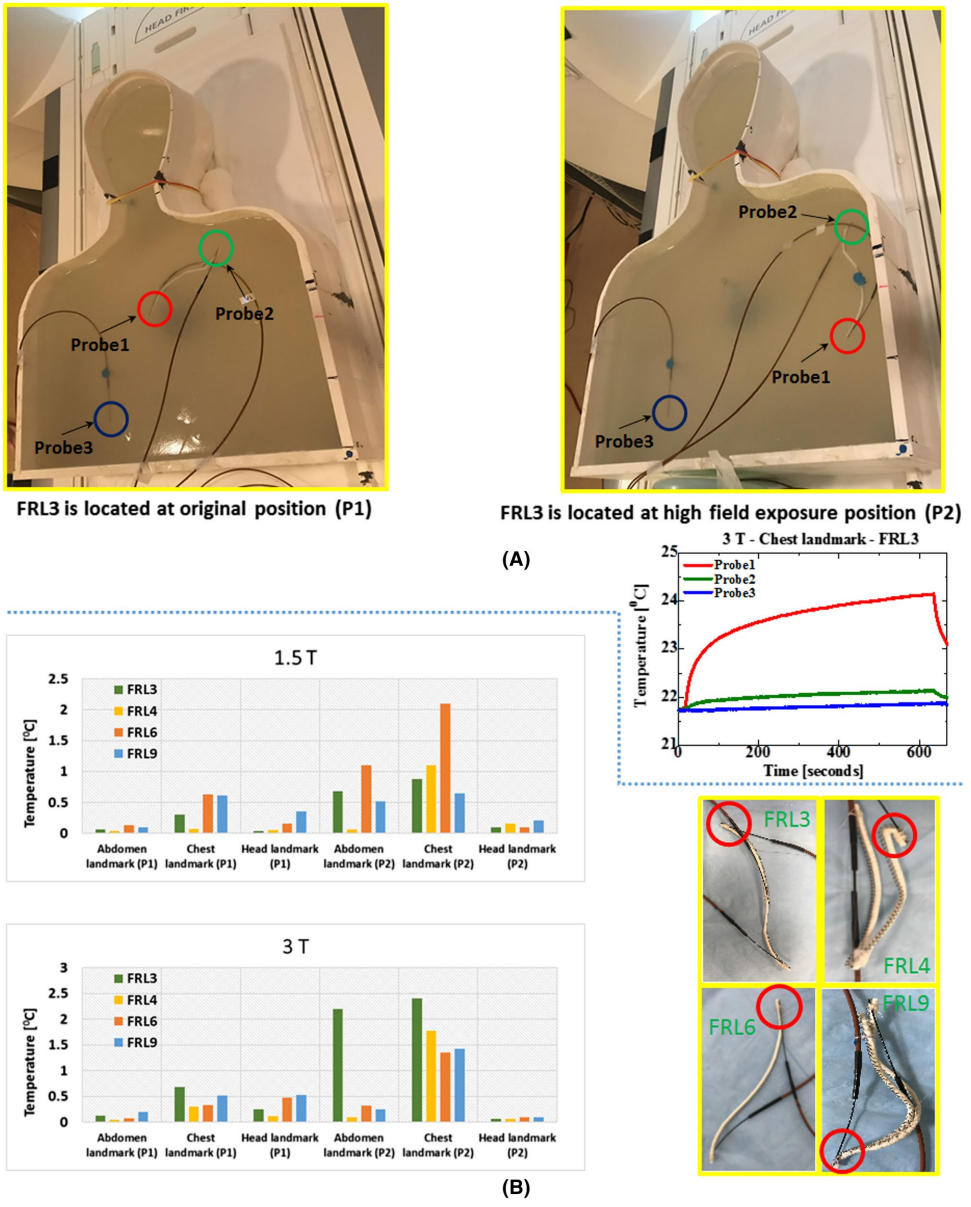
(A) The experimental setup for FRL3 and the positions of temperature probes, as well as their temperature responses at 3 T at chest landmark in the P2 case. (B) Maximum temperature rise along length of FRL models at 1.5 T and 3 T, as well as the locations where the maximum temperatures were measured

Figure 6B gives the maximum Δ*T* recorded along the length of each FRL model at each landmark and field strength. Imaging at chest landmark produced highest heating at both 1.5 T and 3 T, consistent with simulation predictions. In addition, higher heating was observed at 3 T compared with 1.5 T for most of the cases, which is also consistent with the simulation results. The maximum Δ*T* at high field exposure position (P2) was 2.4°C for FRL #3 during MRI at 3 T and 2.1°C for FRL #6 during MRI at 1.5 T. The corresponding values of maximum heating for realistic position (P1) were 0.7°C for 3 T and 0.6°C for 1.5 T.

### 3.4 Estimated extrinsic voltages

Table 4 gives the values of gradient‐induced extrinsic potentials calculated from Supporting Information Table S3 (based on table A.3 in ISO‐TS10794) and a conservative estimation of induced E field in the tissue between the two ends of each FRL. The strength‐duration parameters estimated by Reilly^40^ form the basis for the cardiac‐stimulation limit defined in the IEC 60601‐2‐33 safety guidelines.^35^ With a rheobase of 6.2 V/m and a time constant of 3 ms, Reilly calculated the cardiac‐stimulation limit as

$$E<\frac{6.2V/m}{1‐\exp\left(‐\frac{\tau_{\text{eff}}}{3\;\text{ms}}\right)},$$

where *τ*_eff_ is the effective length of the stimulation, which in this case is the rise time of the gradient pulse. Most clinical scanners have a gradient rise time of 0.1 ms, leading to *E* field threshold of 189 V/m. As indicated in Table 4, all induced *E* values remained below this threshold.

**TABLE 4:** Gradient‐induced extrinsic potential

FRL #	Length (cm)	*V*_emf_ (V)	*E*_tissue_ (V/m)
1	24.0	2.84	23.68
2	14.2	1.97	16.44
3	13.4	1.90	12.68
4	18.0	2.31	76.99
5	17.2	2.24	17.22
6	15.5	2.09	13.92
7	2.0	0.32	64.00
8	4.5	0.72	16.74
9	24.6	2.90	24.13
10	8.5	1.36	17.44

## 4 DISCUSSION AND CONCLUSIONS

There is a steady growth in the use of CIEDs in the United States and globally. The trend is likely to continue, with new indications for use and technological advancements in device manufacturing.^43^, ^44^ Despite the increasing need, MRI is still largely inaccessible to patients with CIEDs because of safety hazards underscored by several injuries reported worldwide.^45^, ^46^, ^47^, ^48^ Although new generations of pacemakers and defibrillators have reduced safety risks associated with MRI static and gradient fields affecting device function, tissue heating from the RF excitation fields remain a major issue. This so‐called “antenna effect” happens when the electric field of the MRI transmitter couples with implanted leads of the CIED, causing the SAR of the RF energy to significantly amplify at the implant’s tip. In vitro and in vivo studies report temperature rises up to 20°C at the lead tip, highlighting the significance of the issue.^49^, ^50^

Efforts to develop MRI‐compatible CIEDs are recent, with newly approved devices allowing conditional MRI of patients with intact cardiac leads at both 1.5 T and 3 T scanners. There is, however, a sizeable cohort of patients with abandoned or retained leads for whom MRI under current labeling is an absolute contraindication, mostly because very little is known about the phenomenology of MRI‐induced heating in the tissue in the presence of partially extracted leads. The major challenge in quantifying implant‐induced heating in this case is that the problem has a large parameter space with many variables that interact with each other. This includes the frequency and geometry of MRI RF coil, the length, trajectory, and structure of the abandoned/retained lead, the imaging landmark, and the patient’s anatomy. Such complexity precludes the application of a systematic experimental approach to infer the worst‐case heating scenario. Numerical simulations, on the other hand, provide an exquisite methodology for exploring thousands of variable combinations in a holistic manner, allowing analysis of parameter extremes outside the bounds of normal clinical practice. This work provides the first comprehensive simulation study of MRI hazards in patients with FRLs, with a focus on RF heating and unintended tissue stimulation. We performed a total of 1800 simulations with 10 patient‐derived FRL models registered to body models with a range of low to high relative permittivities (ε*_r_* = 40–80) and conductivities (σ = 0.1–1.0 *S*/*m*), positioned in MRI RF coils tuned to 64 and 127 MHz, and at different imaging landmarks corresponding to head, chest, and abdomen imaging. In general, body models with lower conductivity generated higher SAR around FRL models. The trend was specifically pronounced at 3 T, where the maximum of SAR1g was almost always generated in the body model with σ = 0.1 *S*/*m* (with the exception of FRL #2 and FRL #7 at chest landmark, where body model with σ = 0.2 *S*/*m* and σ = 0.6 *S*/*m* generated the maximum SAR, respectively).

In terms of imaging landmark, we found agreement in both simulation and experiment results that chest landmark produced significantly higher SAR compared with head and abdomen landmarks at both 1.5 T and 3 T. Specifically, at 1.5 T, MaxSAR1g was about four‐fold higher during RF exposure at chest landmark compared with head and abdomen landmarks. Similarly, at 3 T, MaxSAR1g was about three‐fold and five‐fold higher at chest landmark compared with head and abdomen landmarks, respectively. This was predictable, considering that FRLs were almost exclusively located in the subclavian vein, which would position them at the location of maximum RF field for the coil iso‐center at chest. The experimental measurements predicted similar heating pattern in terms of variation in imaging landmarks and RF frequency. Radiofrequency heating at both 1.5 T and 3 T MRI remained below 3°C for all of the FRL models used in the measurement. However, it should be noted that only one lead type (Medtronic 5076) was used for the experiments. Other lead types should be included in future studies for validation.

Although SAR has been used as a surrogate to assess risks of RF heating of implants, temperature rise in the tissue is the ultimate indicator of tissue damage. Here we used a highly conservative approach to calculate the temperature rise Δ*T* in the tissue around each FRL model by (1) using the body model that generated the maximum SAR around each FRL model, (2) eliminating cooling effects of perfusion from Penn’s bioheat equation, and (3) calculating the Δ*T* for a continuous RF exposure, despite the fact that almost all MRI sequences have duty cycles well below 100%. Another level of conservatism was applied in determination of CEM_43_, which was calculated using Δ*T* from the FRL model and produced the maximum RF heating. The RF exposure with B_1_^+^ ≤ 2 µT generated CEM_43_ < 40 at all imaging landmarks, indicating no thermal damage for acquisition times <10 min. It should be noted, however, that many clinical sequences with higher B_1_^+^ are much shorter than 10 min, and thus generate negligible CEM_43_. Finally, a highly conservative assessment of electric field induced in the tissue due to gradient‐induced voltages induced along the length of the FRLs suggested that the risk of unintended tissue stimulation was negligible.

Furthermore, we would like to emphasize the difference between “abandoned” and “retained” leads. Although the words are sometimes used interchangeably, they refer to substantially different scenarios in terms of MRI RF heating. Abandoned leads are leads that are simply disconnected from the CIED generator and left in the body. As such, their internal geometry and trajectory remains intact, which means conductive wire remain within the insulation with only the tip exposed to the tissue. Retained leads, on the other hand, are fragments of bare conductive wires (the insulation comes off during the extraction) that are left in the body after an attempt is made to extract the lead. As RF heating of insulated leads is generally higher than RF heating of bare leads, the results of this study should not be generalized to assume safety of MRI in patients with abandoned leads or any FRL lead that has insulation retained with it.

Finally, although MRI is currently contraindicated in patients with FRLs, recent studies that retrospectively analyzed available radiographic images and clinical records found no adverse effects (AEs) associated with application of MRI in patients with FRLs.^51^ Our work provides theoretical and experimental evidence that MRI, under certain conditions (i.e., for RMS B_1_^+^ ≤ 2 µT) may be performed safely in patients with FRLs, and as such can serve as a guideline for future applications. These findings suggest that in patients with FRLs, whom their clinical management would be changed based on the diagnostic power of MRI, and there is no alternative to MRI such as CT imaging, MRI with caution may be performed without substantial risk for an AE; however, for routine use of MRI in these patients, a larger cohort of patients must be studied.

## Supporting information


**FIGURE S1** Maximum value of 1*g*‐averaged specific absorption rate (MaxSAR1g) generated around retained lead models fragmented retained lead 1 (FRL1) and FRL2 as a function of body model’s conductivity (horizontal axis) and permittivity (different colored graphs). The field strength and imaging landmark are noted on top of each plot
**FIGURE S2** (1) Two ends of the FRLs. (2) Tangential component of gradient‐induced electric field along the FRL. A conservative estimation of the induced voltage Vemf along the FRL was calculated by multiplying the maximum value of gradient‐induced E field (based on simulations given in Annex B of ISO‐TS 10974) by the FRL length. (3) Gradient field. (4) Conservative estimation of E field in the tissue was calculated by dividing Vemf by the distance between two ends of the lead
**FIGURE S3** The MaxSAR1g generated around retained lead models FRL3 and FRL4 as a function of body model’s conductivity (horizontal axis) and permittivity (different colored graphs). The field strength and imaging landmark are noted on top of each plot
**FIGURE S4** The MaxSAR1g generated around retained lead models FRL5 and FRL6 as a function of body model’s conductivity (horizontal axis) and permittivity (different colored graphs). The field strength and imaging landmark are noted on top of each plot
**FIGURE S5** The MaxSAR1g generated around retained lead models FRL7 and FRL8 as a function of the body model’s conductivity (horizontal axis) and permittivity (different colored graphs). The field strength and imaging landmark are noted on top of each plot
**FIGURE S6** The MaxSAR1g generated around retained lead models FRL9 and FRL10 as a function of body model’s conductivity (horizontal axis) and permittivity (different colored graphs). The field strength and imaging landmark are noted on top of each plot
**FIGURE S7** Measured temperature rise along length of 20 cm wire at 1.5 T. The wire was located at the left and the right of the phantom, and the depth of the wire was 2, 5, and 8 cm from the top of the gel (maximum thickness = 12 cm)
**TABLE S1** Temperature rise Δ*T* (°C) in the tissue surrounding the fragmented retained leads after 10 minutes of continuous RF exposure at 64 MHz (1.5 T) for the coil iso‐center positioned at different imaging landmarks and the input power adjusted to generate different B_1_^+^ values on an axial plane passing through the center of the coil
**TABLE S2** Temperature rise Δ*T* (°C) in the tissue surrounding the fragmented retained leads after 10 minutes of continuous RF exposure at 127 MHz (3 T) for the coil iso‐center positioned at different imaging landmarks and the input power adjusted to generate different B_1_^+^ values on an axial plane passing through the center of the coil
**TABLE S3** Lead length factor L
