# Supplementary material for: Safety of MRI in patients with retained cardiac leads
Source: Magn Reson Med. 2021 Dec 27;87(5):2464–80. doi: 10.1002/mrm.29116 (PMC8919805; doi:10.1002/mrm.29116)
Supplement: Supplementary file 1 — FIGURE S1 Maximum value of 1g‐averaged specific absorption rate (MaxSAR1g) generated around retained lead models fragmented retained lead 1 (FRL1) and FRL2 as a function of body model’s conductivity (horizontal axis) and permittivity (different colored graphs). The field strength and imaging landmark are noted on top of each plot FIGURE S2 (1) Two ends of the FRLs. (2) Tangential component of gradient‐induced electric field along the FRL. A conservative estimation of the induced voltage Vemf along the FRL was calculated by multiplying the maximum value of gradient‐induced E field (based on simulations given in Annex B of ISO‐TS 10974) by the FRL length. (3) Gradient field. (4) Conservative estimation of E field in the tissue was calculated by dividing Vemf by the distance between two ends of the lead FIGURE S3 The MaxSAR1g generated around retained lead models FRL3 and FRL4 as a function of body model’s conductivity (horizontal axis) and permittivity (different colored graphs). The field strength and imaging landmark are noted on top of each plot FIGURE S4 The MaxSAR1g generated around retained lead models FRL5 and FRL6 as a function of body model’s conductivity (horizontal axis) and permittivity (different colored graphs). The field strength and imaging landmark are noted on top of each plot FIGURE S5 The MaxSAR1g generated around retained lead models FRL7 and FRL8 as a function of the body model’s conductivity (horizontal axis) and permittivity (different colored graphs). The field strength and imaging landmark are noted on top of each plot FIGURE S6 The MaxSAR1g generated around retained lead models FRL9 and FRL10 as a function of body model’s conductivity (horizontal axis) and permittivity (different colored graphs). The field strength and imaging landmark are noted on top of each plot FIGURE S7 Measured temperature rise along length of 20 cm wire at 1.5 T. The wire was located at the left and the right of the phantom, and the depth of the wire was 2, 5, and 8 c [file MRM-87-2464-s001.docx]

**Supplemental Materials**


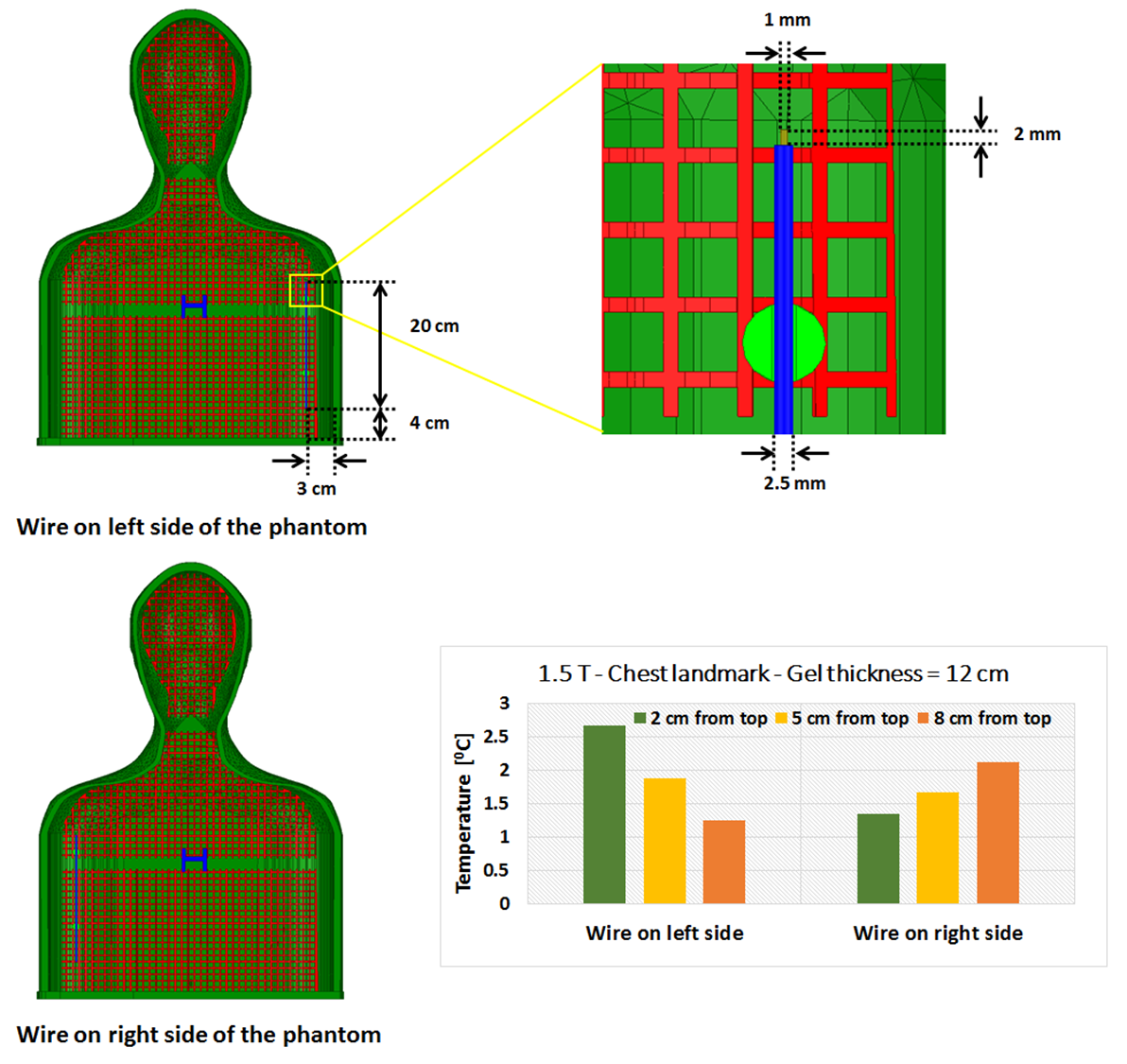


Supporting Information Figure S1. Measured temperature rise along length of 20 cm wire at 1.5 T. The wire was located at the left and the right of the phantom, and the depth of the wire was 2 cm, 5 cm and 8 cm from the top of the gel (maximum thickness = 12 cm).


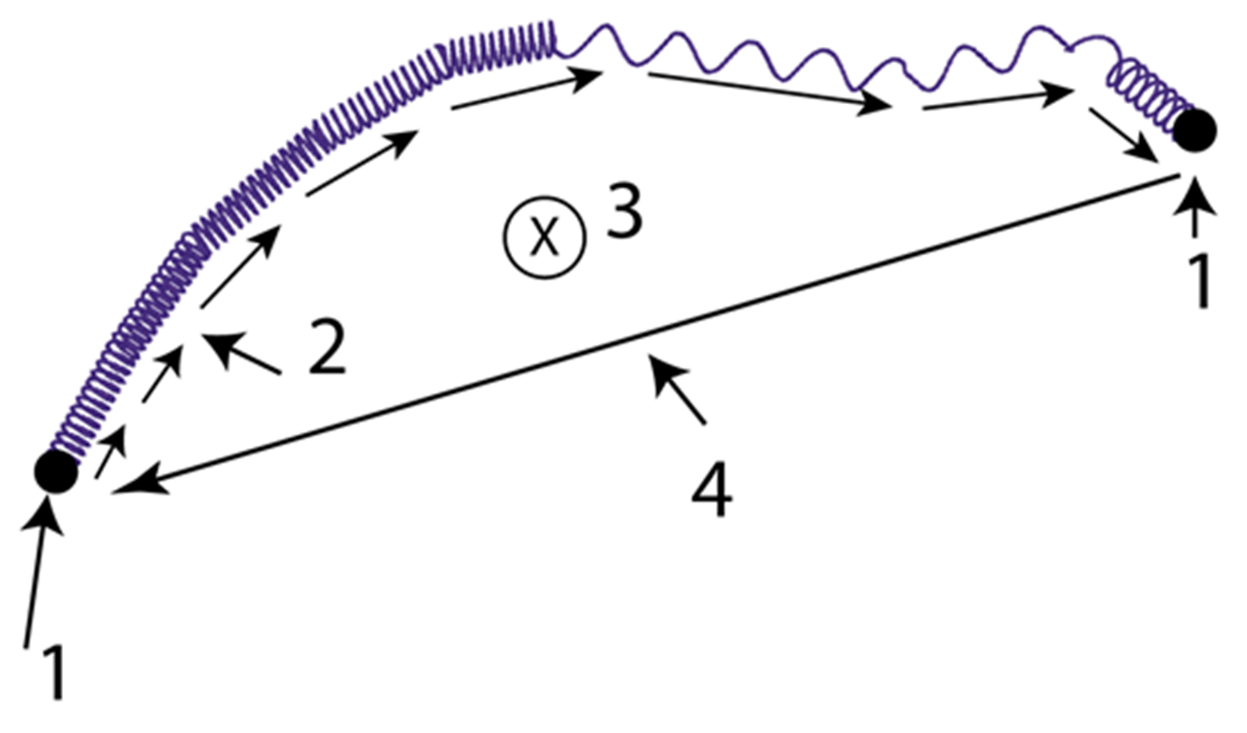


Supporting Information Figure S2. (1): Two ends of the FRL. (2) Tangential component of gradient-induced electric field along the FRL. A conservative estimation of the induced voltage V_emf_ along the FRL was calculated by multiplying the maximum value of gradient-induced E field (based on simulations given in Annex B of ISO-TS 10974) by the FRL length. (3) Gradient field (4) A conservative estimation of E field in the tissue was calculated by dividing V_emf_ by the distance between two ends of the lead.

**
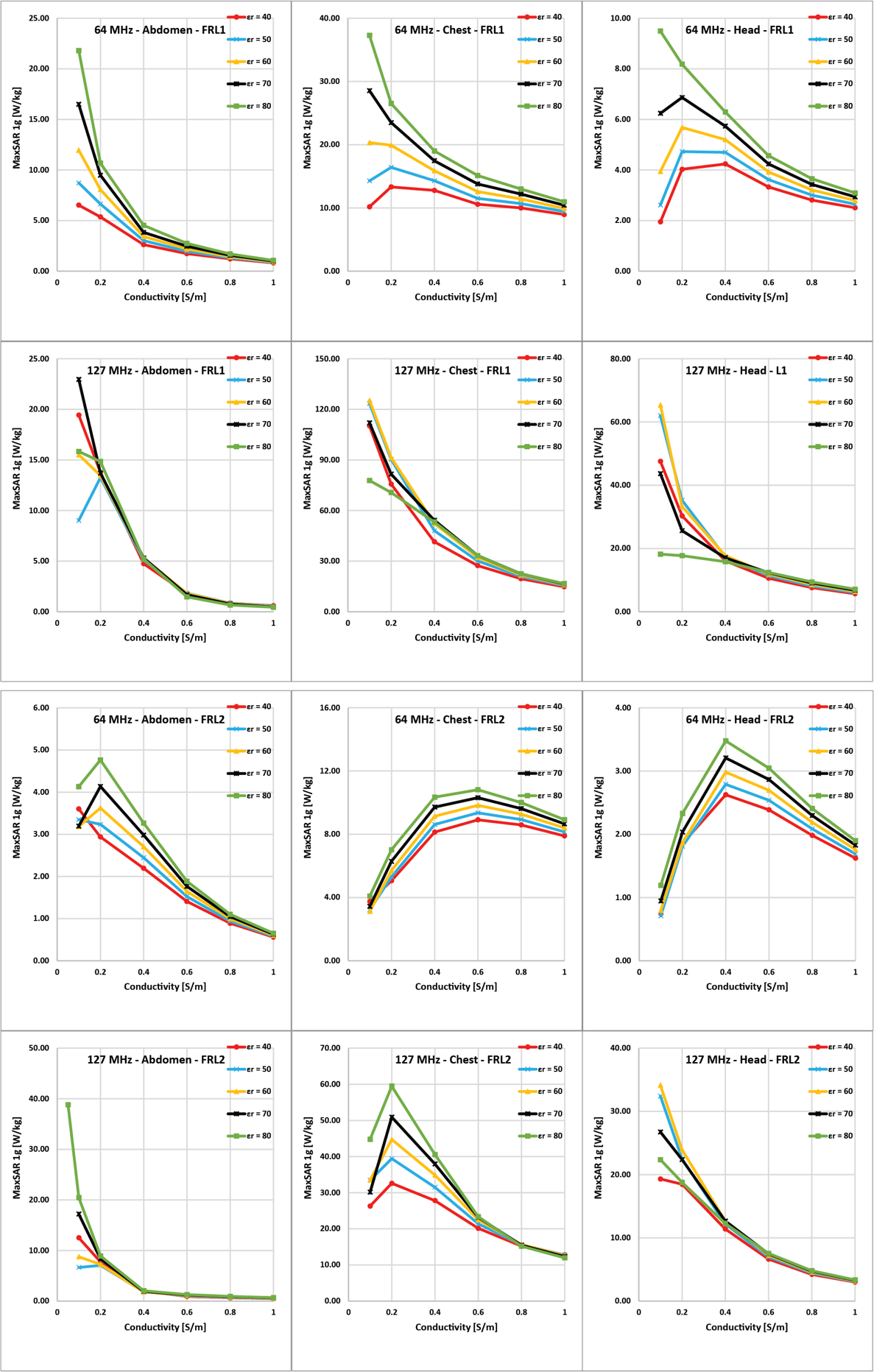
**

Supporting Information Figure S3. MaxSAR1g generated around retained lead models FRL1 and FRL2 as a function of body model’s conductivity (horizontal axis) and permittivity (different-colored graphs). The field strength and imaging landmark is noted on top of each plot.

**
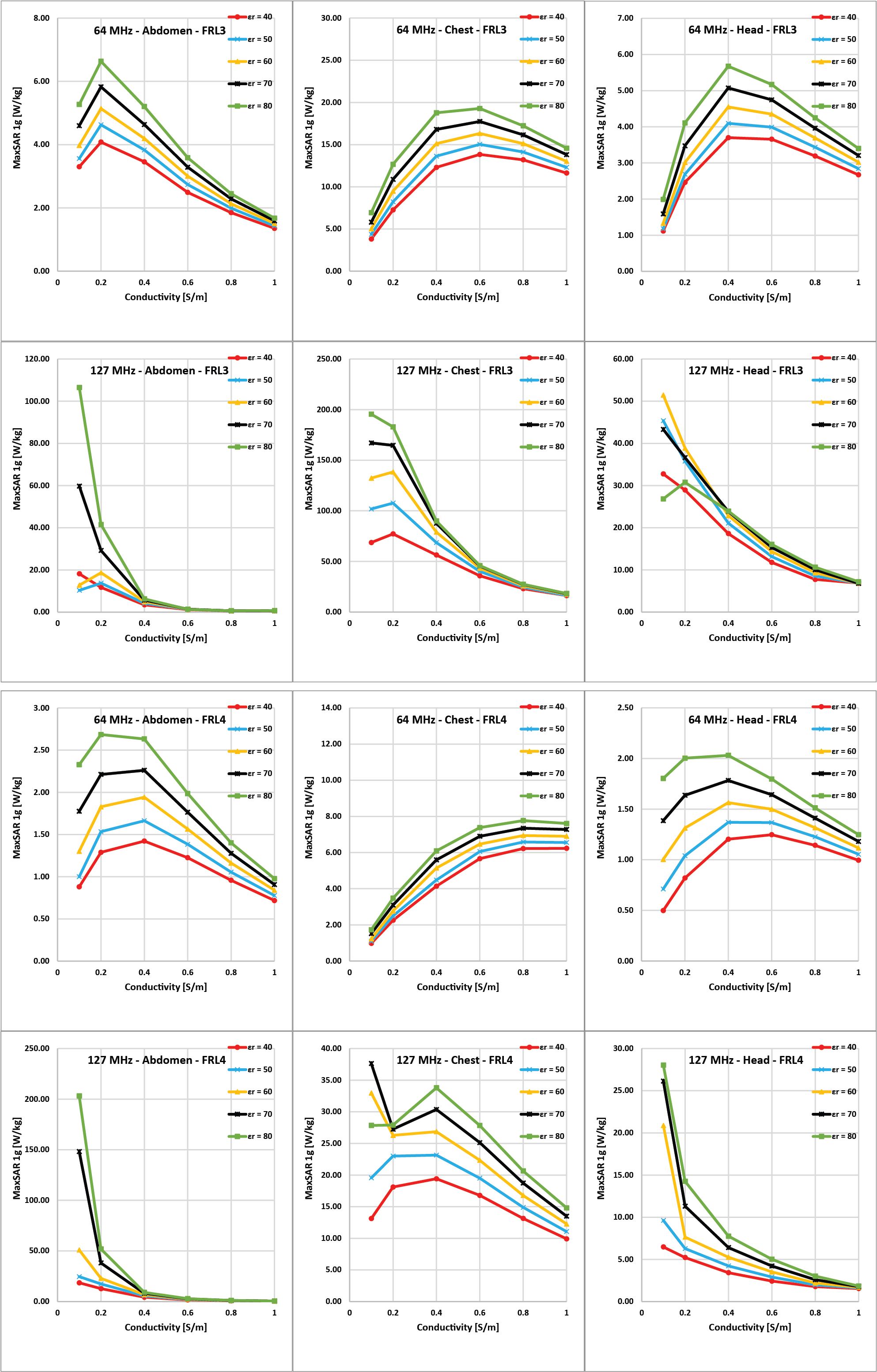
**

Supporting Information Figure S4. MaxSAR1g generated around retained lead models FRL3 and FRL4 as a function of body model’s conductivity (horizontal axis) and permittivity (different-colored graphs). The field strength and imaging landmark is noted on top of each plot.

**
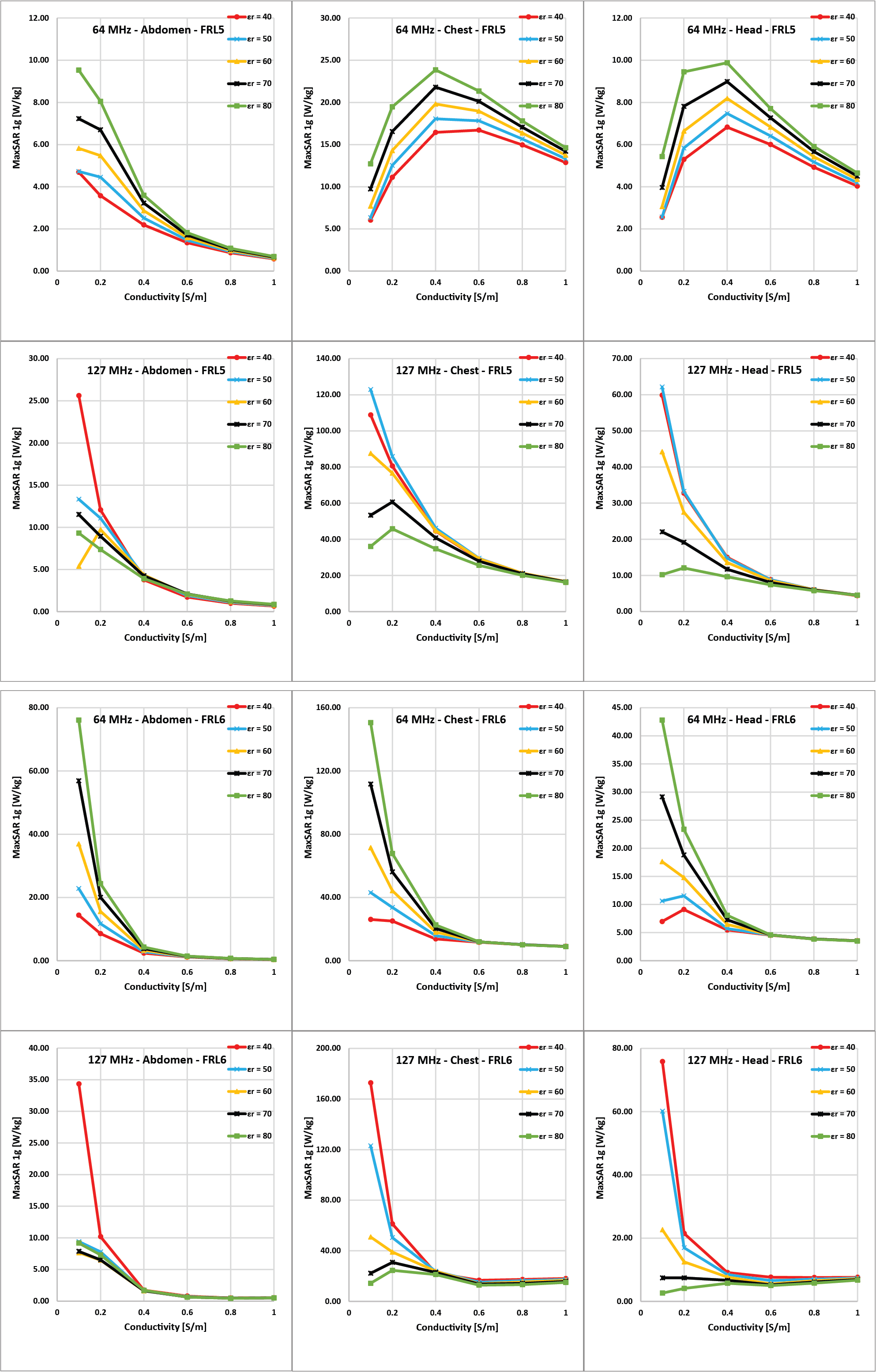
**

Supporting Information Figure S5. MaxSAR1g generated around retained lead models FRL5 and FRL6 as a function of body model’s conductivity (horizontal axis) and permittivity (different-colored graphs). The field strength and imaging landmark is noted on top of each plot.

**
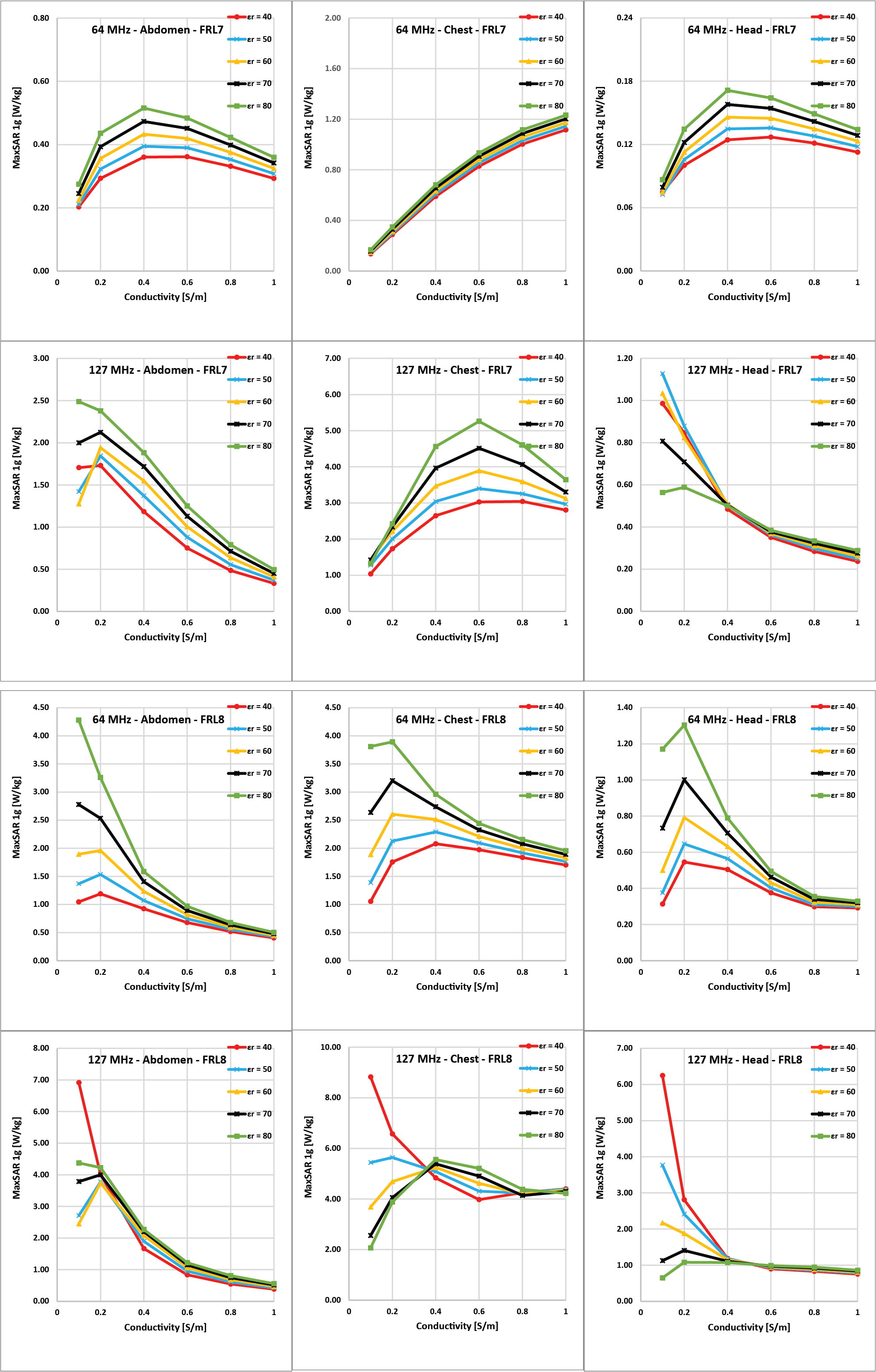
**

Supporting Information Figure S6. MaxSAR1g generated around retained lead models FRL7 and FRL8 as a function of body model’s conductivity (horizontal axis) and permittivity (different-colored graphs). The field strength and imaging landmark is noted on top of each plot.

**
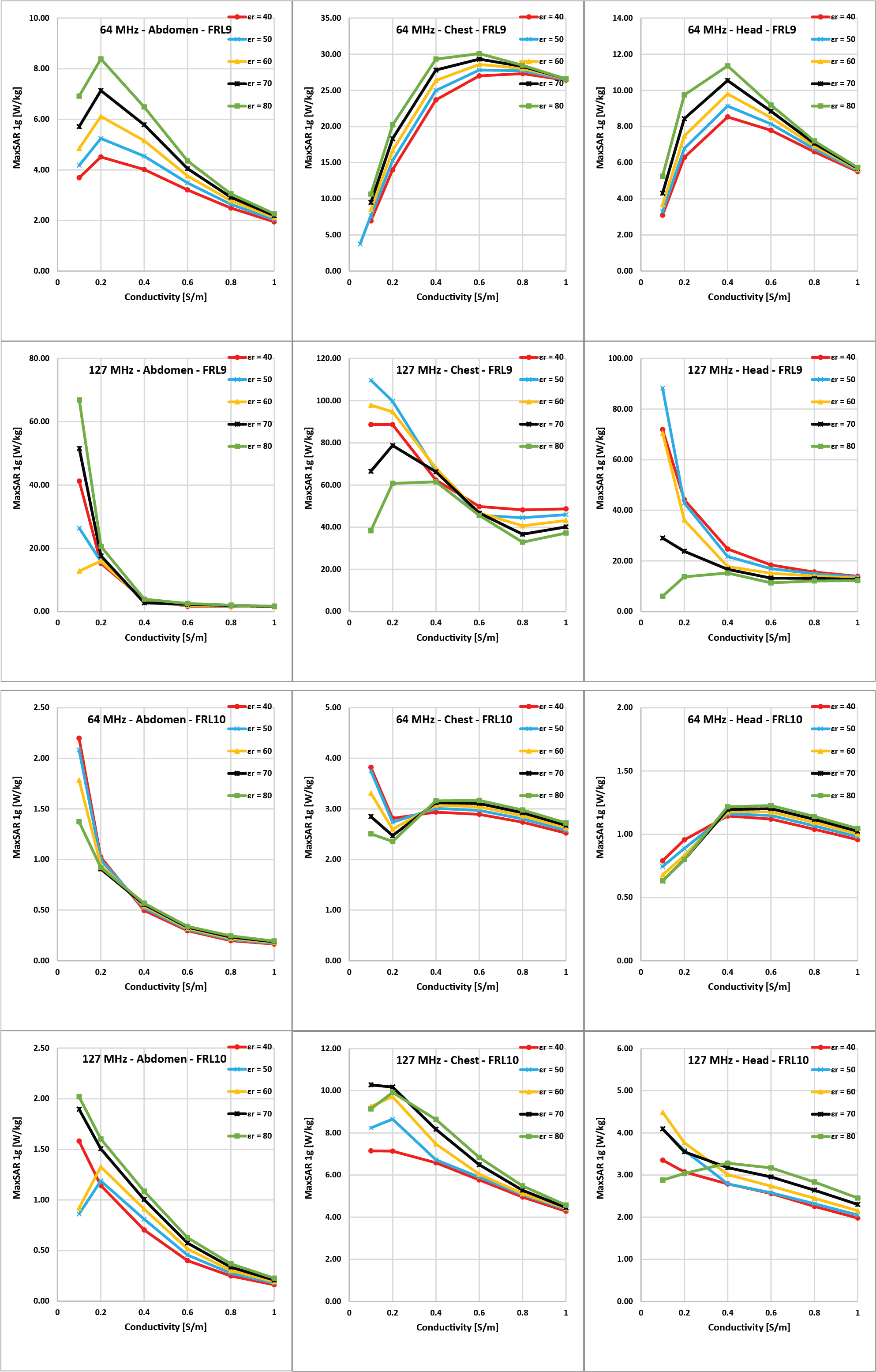
**

Supporting Information Figure S7. MaxSAR1g generated around retained lead models FRL9 and FRL10 as a function of body model’s conductivity (horizontal axis) and permittivity (different-colored graphs). The field strength and imaging landmark is noted on top of each plot.

Supporting Information Table S1. Temperature rise $\Delta T [℃]$ in the tissue surrounding the FRL after 10-minute continuous RF exposure at 64 MHz (1.5 T) for the coil iso-center positioned at different imaging landmarks and the input power adjusted to generate different B_1_^+^ values on an axial plane passing through center of the coil.

| FRL # | B_1_^+^ [$\mu T$] | | | | | Landmark |
| --- | --- | --- | --- | --- | --- | --- |
|  | 1 | 2 | 3 | 4 | 5 |  |
| 1 | 0.14 | 0.57 | 1.28 | 2.27 | 3.55 | Abdomen |
|  | 0.23 | 0.94 | 2.11 | 3.75 | 5.87 | Chest |
|  | 0.09 | 0.37 | 0.84 | 1.49 | 2.32 | Head |
| 2 | 0.05 | 0.22 | 0.49 | 0.87 | 1.36 | Abdomen |
|  | 0.18 | 0.74 | 1.66 | 2.95 | 4.61 | Chest |
|  | 0.10 | 0.42 | 0.95 | 1.69 | 2.64 | Head |
| 3 | 0.12 | 0.49 | 1.10 | 1.96 | 3.06 | Abdomen |
|  | 0.23 | 0.70 | 2.11 | 3.76 | 5.87 | Chest |
|  | 0.11 | 0.43 | 0.96 | 1.70 | 2.66 | Head |
| 4 | 0.10 | 0.41 | 0.91 | 1.62 | 2.53 | Abdomen |
|  | 0.26 | 1.02 | 2.30 | 4.09 | 6.38 | Chest |
|  | 0.11 | 0.42 | 0.96 | 1.70 | 2.66 | Head |
| 5 | 0.08 | 0.31 | 0.70 | 1.24 | 1.94 | Abdomen |
|  | 0.29 | 1.16 | 2.60 | 4.62 | 7.22 | Chest |
|  | 0.14 | 0.55 | 1.23 | 2.18 | 3.40 | Head |
| 6 | 0.94 | 3.76 | 8.46 | 15.04 | 23.50 | Abdomen |
|  | 2.01 | 8.03 | 18.06 | 32.12 | 50.18 | Chest |
|  | 0.53 | 2.14 | 4.81 | 8.55 | 13.35 | Head |
| 7 | 0.16 | 0.65 | 1.45 | 2.58 | 4.03 | Abdomen |
|  | 0.40 | 1.59 | 3.57 | 6.35 | 9.93 | Chest |
|  | 0.03 | 0.44 | 0.98 | 1.74 | 2.72 | Head |
| 8 | 0.06 | 0.23 | 0.52 | 0.93 | 1.46 | Abdomen |
|  | 0.05 | 0.20 | 0.45 | 0.81 | 1.26 | Chest |
|  | 0.06 | 0.26 | 0.57 | 1.02 | 1.60 | Head |
| 9 | 0.10 | 0.39 | 0.89 | 1.58 | 2.46 | Abdomen |
|  | 0.43 | 1.73 | 3.90 | 6.94 | 10.84 | Chest |
|  | 0.14 | 0.56 | 1.25 | 2.23 | 3.48 | Head |
| 10 | 0.05 | 0.22 | 0.48 | 0.86 | 1.34 | Abdomen |
|  | 0.04 | 0.14 | 0.32 | 0.57 | 0.90 | Chest |
|  | 0.14 | 0.57 | 1.27 | 2.26 | 3.54 | Head |

Supporting Information Table S2. Temperature rise $\Delta T [℃]$ in the tissue surrounding the FRL after 10-minute continuous RF exposure at 127 MHz (3 T) for the coil iso-center positioned at different imaging landmarks and the input power adjusted to generate different B_1_^+^ values on an axial plane passing through center of the coil.

| FRL # | B_1_^+^ [$\mu T$] | | | | | Landmark |
| --- | --- | --- | --- | --- | --- | --- |
|  | 1 | 2 | 3 | 4 | 5 |  |
| 1 | 0.26 | 1.02 | 2.30 | 4.09 | 6.39 | Abdomen |
|  | 1.25 | 4.98 | 11.20 | 19.91 | 31.11 | Chest |
|  | 1.16 | 4.64 | 10.44 | 18.55 | 28.99 | Head |
| 2 | 0.26 | 1.03 | 2.32 | 4.13 | 6.46 | Abdomen |
|  | 0.42 | 1.67 | 3.76 | 6.69 | 10.45 | Chest |
|  | 0.37 | 1.49 | 3.35 | 5.95 | 9.30 | Head |
| 3 | 1.94 | 7.74 | 17.42 | 30.97 | 48.40 | Abdomen |
|  | 2.46 | 9.82 | 22.10 | 39.29 | 61.39 | Chest |
|  | 0.78 | 3.13 | 7.04 | 12.51 | 12.97 | Head |
| 4 | 2.24 | 8.94 | 20.12 | 35.77 | 55.90 | Abdomen |
|  | 0.28 | 1.12 | 2.52 | 4.48 | 6.99 | Chest |
|  | 0.26 | 1.03 | 2.31 | 4.10 | 6.41 | Head |
| 5 | 0.28 | 2.07 | 2.55 | 4.53 | 7.08 | Abdomen |
|  | 1.43 | 5.70 | 12.83 | 22.81 | 35.65 | Chest |
|  | 0.53 | 2.11 | 4.75 | 8.45 | 13.21 | Head |
| 6 | 0.37 | 1.47 | 3.32 | 5.90 | 9.21 | Abdomen |
|  | 2.07 | 8.27 | 18.62 | 33.09 | 51.71 | Chest |
|  | 0.73 | 2.94 | 6.61 | 11.75 | 18.37 | Head |
| 7 | 0.27 | 1.07 | 2.30 | 4.28 | 6.68 | Abdomen |
|  | 1.18 | 4.72 | 10.62 | 18.89 | 29.51 | Chest |
|  | 0.13 | 0.51 | 1.15 | 2.05 | 3.20 | Head |
| 8 | 0.22 | 0.88 | 1.98 | 3.51 | 5.49 | Abdomen |
|  | 0.07 | 0.28 | 0.63 | 1.12 | 1.74 | Chest |
|  | 0.13 | 0.53 | 1.20 | 2.13 | 3.32 | Head |
| 9 | 0.81 | 3.24 | 7.29 | 12.96 | 20.25 | Abdomen |
|  | 1.53 | 6.11 | 13.75 | 24.44 | 38.19 | Chest |
|  | 0.99 | 3.98 | 8.96 | 15.93 | 24.88 | Head |
| 10 | 0.27 | 1.09 | 2.44 | 4.34 | 6.78 | Abdomen |
|  | 0.09 | 0.37 | 0.83 | 1.47 | 2.30 | Chest |
|  | 0.10 | 0.40 | 0.90 | 1.60 | 2.49 | Head |

Supporting Information Table S3. Lead length factor L.

| Lead length [cm] | V_emf_ [Volt] |
| --- | --- |
| $l\leq10$ | $16\times{10}^{-2}\times l$ |
| $10<l<63$ | $8.87\times{10}^{-2}\times l-7.13\times{10}^{-1}$ |
